# Lipid and Alzheimer’s disease genes associated with healthy aging and longevity in healthy oldest-old

**DOI:** 10.18632/oncotarget.15296

**Published:** 2017-02-11

**Authors:** Lauren C. Tindale, Stephen Leach, John J. Spinelli, Angela R. Brooks-Wilson

**Affiliations:** ^1^ Canada’s Michael Smith Genome Sciences Centre, British Columbia Cancer Agency, Vancouver, B.C., Canada; ^2^ Department of Biomedical Physiology and Kinesiology, Simon Fraser University, Burnaby, B.C., Canada; ^3^ Cancer Control Research, British Columbia Cancer Agency, Vancouver, B.C., Canada; ^4^ School of Population and Public Health, University of British Columbia, Vancouver, B.C., Canada

**Keywords:** healthy aging, longevity, Alzheimer’s, disease, APOE, buffering, epistasis, Gerotarget

## Abstract

Several studies have found that long-lived individuals do not appear to carry lower numbers of common disease-associated variants than ordinary people; it has been hypothesized that they may instead carry protective variants. An intriguing type of protective variant is buffering variants that protect against variants that have deleterious effects. We genotyped 18 variants in 15 genes related to longevity or healthy aging that had been previously reported as having a gene-gene interaction or buffering effect. We compared a group of 446 healthy oldest-old ‘Super-Seniors’ (individuals 85 or older who have never been diagnosed with cancer, cardiovascular disease, dementia, diabetes or major pulmonary disease) to 421 random population-based midlife controls. Cases and controls were of European ancestry. Association tests of individual SNPs showed that Super-Seniors were less likely than controls to carry an *APOEε4* allele or a haptoglobin *HP2* allele. Interactions between *APOE/FOXO3*, *APOE/CRYL1*, and *LPA/CRYL1* did not remain significant after multiple testing correction. In a network analysis of the candidate genes, lipid and cholesterol metabolism was a common theme. *APOE*, *HP*, and *CRYL1* have all been associated with Alzheimer’s Disease, the pathology of which involves lipid and cholesterol pathways. Age-related changes in lipid and cholesterol maintenance, particularly in the brain, may be central to healthy aging and longevity.

## INTRODUCTION

Healthy aging is the ability to age successfully without succumbing to disease, with an emphasis on healthspan over lifespan [1]. The genetics of healthy aging and longevity is complex, with few genetic associations replicating between studies. *APOE* (apolipoprotein E) is an exception; genetic variation in this gene has been associated with longevity in multiple genome-wide association studies (GWAS) and candidate gene studies [1, 2]. The *APOEε4* allele is associated with increased mortality, and is also the major genetic risk factor for late onset Alzheimer's disease (AD). While the *ε4* allele is neither necessary nor sufficient for developing the disease, it increases risk in a dose-dependent manner [3].

Long-lived individuals have been found to carry a burden of disease-associated variants comparable to that observed in typical individuals [4–6]. One possible explanation for their ability to remain in good health to advanced ages, and still carry deleterious variants, is the concept of genetic buffering. Genetic buffering is a type of epistatic interaction in which a favourable genotype attenuates the effect of one or more deleterious variants. In this model, long-lived individuals may carry harmful (buffered) variants without developing disease, as a result of also carrying protective (buffering) variants. In a paper first suggesting the application of buffering to human longevity, Bergman and colleagues used changes in allele frequencies with age to show buffering of a deleterious *LPA* heterozygote by a buffering *CETP VV* genotype [7] in participants in the Longevity Genes Project [8].

We have assembled a list of genetic variants previously reported as having possible epistatic or buffering/buffered effects related to longevity in human studies. We examined these variants in individuals aged 85 years or older who had never been diagnosed with cancer, cardiovascular disease (CVD), diabetes, dementia, or major pulmonary disease; we call them the ‘Super-Seniors’ [9]. These healthy oldest-old were compared to random population-based middle-aged controls. We hypothesize that epistatic interactions, in which longevity-promoting buffering variants protect against the effects of deleterious buffered variants, contribute to the Super-Seniors’ health and longevity.

## RESULTS

### Candidate variants

A search in PubMed of the combinations “epistasis AND aging”, “epistasis AND longevity”, “buffering AND aging”, “buffering AND longevity”, “human”, and “genetics” produced a list of 111 papers of interest. Manual review of the papers and, in some cases, references cited within them, identified 18 variants in 15 genes suspected as having an interaction related to aging or longevity (Table 1). This included 15 SNPs, a 1bp deletion, a 1724bp deletion, and the well-characterized *APOE* haplotype.

**Table 1:** Candidate genes and candidate epistatic variants

Gene	ID	Effect	Proposed Interaction	Reference
*APOA1*	rs670	Deleterious	Buffered	Garasto et al., 2003 [45]
*APOE*	*APOE*ε4	Deleterious	*APOE*ε4 buffered by *HP1*/*1*	Napolioni et al., 2011 [44]
*HFE*	rs1800562	Deleterious	*HFE T* allele buffered	Tan et al., 2003 [24]
*KL*	rs9536314	Deleterious	*KL het* buffered	Bergman et al., 2007 [7]
*LPA*(1)	rs1853021	Deleterious	*LPA het* buffered by *CETP VV*	Bergman et al., 2007 [7]
*LPA*(2)	rs3798220	Deleterious	Risk for coronary disease	Clarke et al., 2009 [22]
*LPA*(3)	rs10455872	Deleterious	Risk for coronary disease	Clarke et al., 2009 [22]
*MTTP*	rs2866164	Deleterious	*MTTP CC* buffered by *APOC3 CC*, *CETP VV*, *ADIPOQ del*/*del*	Huffman et al., 2012 [46]
*PON1*	rs662	Deleterious	*PON1 het* buffered	Bonafè et al., 2002 [47]
*ADIPOQ*	rs56354395	Protective	*ADIPOQ del/del* buffers *MTTP CC*	Atzmon et al., 2008 [27]
*APOC3*	rs595049 (LD with rs2542052)	Protective	*APOC3 CC* buffers *MTTP CC*	Atzmon et al., 2006 [48]
*CETP*	rs5882	Protective	*CETP VV* buffers *MTTP CC*, *LPA*(1) *het*	Barzilai et al., 2003 [29]
*CRYL1*	rs7989332	Protective	AD-associated with *KHDRBS2*	Gusareva et al., 2014 [15]
*FOXO1*	rs2701858	Protective	Joint effect with *FOXO3*(1) for longevity	Tan et al., 2013 [49]
*FOXO3*(1)	rs9486902	Protective	Joint effect with *FOXO1* for longevity	Tan et al., 2013 [49]
*FOXO3*(2)	rs2802292	Protective	*FOXO3 GG* buffering	Willcox et al., 2008 [20]
*HP*	rs72294371	Protective	*HP1*/*1* buffers *APOE*ε4	Napolioni et al., 2011 [44]
*KHDRBS2*	rs6455128	Protective	AD-associated with *CRYL1*	Gusareva et al., 2014 [15]

Effect indicates whether the variant was considered be deleterious or protective in the original literature report. Het = heterozygous, AD = Alzheimer's disease.

### Genotypes and quality control

After excluding 11 samples with a call rate < 90%, there were 459 (152 male, 307 female) Super-Seniors and 417 (166 male, 251 female) controls. The haptoglobin (*HP*) variant genotyped by PCR had a call rate of 93%. SNP call rates all exceeded 95%. *LPA* SNP rs3798220 had a minor allele frequency (MAF) < 5% in our study population so was excluded from analysis. There were no significant deviations from Hardy-Weinberg Equilibrium in controls when corrected using false discovery rate.

### Association tests of individual variants

There was a greater proportion of female Super-Seniors [odds ratio (OR) 1.33, 95% confidence interval (CI) = 1.01-1.76], so sex was included in all models. Genotype frequencies for all variants are shown in Table 2. When the 17 variants were tested for association with healthy aging, under dominant and additive models, only the *HP* and *APOE* variants showed significant associations (Table 3 and Table S1).

**Table 2:** Genotype counts and frequencies in Super-Seniors and controls

						Super-Seniors			Controls		
Gene	ID	Alleles*	MAF in study	MAF in 1000 Genomes	GRGh38 genomic location	Homo major allele	Het	Homo minor allele	Homo major allele	Het	Homo minor allele
*ADIPOQ*	rs56354395	A>del	0.370	0.499	3:186855076/5	182	212	54	159	192	63
*APOA1*	rs670	C>T	0.158	0.188	11:116837697	308	123	8	283	110	8
*APOC3*	rs595049	T>G	0.345	0.498	11:116828729	204	196	59	176	191	50
*APOE*	*APOE*ε4	ε2/ ε3>ε4	0.128			365	84	4	293	109	10
*CETP*	rs5882	T>C	0.304	0.466	16:56982180	209	190	44	198	171	32
*CRYL1*	rs7989332	G>T	0.261	0.222	13:20476436	249	179	30	224	169	24
*FOXO1*	rs2701858	G>A	0.065	0.108	13:40564252	388	63	2	371	41	2
*FOXO3*	rs9486902	C>T	0.142	0.174	6:108556849	341	100	13	305	97	12
*FOXO3*	rs2802292	A>C	0.366	0.469	6:108587315	162	226	55	166	189	47
*HFE*	rs1800562	C>T	0.067	0.013	6:26092913	394	64	1	367	49	1
*HP*	rs72294371	*HP*2>*HP*1	0.448			126	199	99	123	202	63
*KHDRBS2*	rs6455128	C>A	0.178	0.219	6:61987841	321	117	21	279	122	16
*KL*	rs9536314	T>G	0.163	0.130	13:33054001	334	114	11	283	116	17
*LPA*	rs1853021	C>T	0.152	n/a	6:160664263	324	119	9	303	96	15
*LPA*	rs3798220	T>C	0.017	0.051	6:160540105	445	14	0	402	15	0
*LPA*	rs10455872	T>C	0.070	0.022	6:160589086	403	54	2	357	56	4
*MTTP*	rs2866164	C>G	0.256	0.250	4:99569786	234	168	30	229	137	29
*PON1*	rs662	A>G	0.284	0.457	7:95308134	228	177	38	212	151	37

^*^Major allele > minor allele. Minor allele frequency (MAF) was calculated from the entire study population.

**Table 3:** Odds ratios and 95% confidence intervals for the association between variants in *APOE* and *HP* and healthy aging

Variant	Model	Super-Seniors	Controls	Genotype	Odds ratio (95% CI)	*p* value
HP rs72294371	Dominant	99	63	1/1	1	0.010
		325	325	1/2 or 2/2	0.63(0.44-0.90)	(df=1)
	Additive	99	63	1/1	1	0.056 (df=1)
		199	202	1/2	0.62 (0.43-0.90)	
		126	123	2/2	0.64 (0.42-0.96)	
*APOE* haplotype	ε4 Dominant	365 88	293 119	Non- ε4 carrier ε4 carrier	1 0.59 (0.43-0.81)	0.0010 (df=1)
	Overall	283	248	ε3/ε3	1	0.00017 (df=5)
		18	3	ε2/ε2	5.33 (1.55-18.34)	
		64	42	ε2/ε3	1.32 (0.86-2.02)	
		77	94	ε3/ε4	0.71 (0.50-1.01)	
		7	15	ε2/ε4	0.40 (0.16-1.00)	
		4	10	ε4/ε4	0.35 (0.11-1.12)	

Super-Seniors were less likely than controls to carry the known disease risk alleles *HP2* or *APOEε4*. Carriers of the *HP2* allele had decreased odds of being a Super-Senior, OR 0.63 (95% CI = 0.44-0.90, *p* = 0.010), as did *APOEε4* allele carriers, OR 0.59 (95% CI = 0.43-0.81, *p* = 0.0010). The significance of the association with HP did not hold under application of the false discovery rate (FDR) (threshold = 0.05 for 17 comparisons), but *APOE* remained significant after FDR, *p* = 0.017.

Using an additive model, *HP* genotype was associated with healthy longevity with a per allele OR of 0.83 (95% CI = 0.68-1.00, *p* = 0.056). Compared to *HP1* homozygotes, heterozygotes had an OR of 0.62 (95% CI = 0.43-0.90) and *HP2* homozygotes had an OR of 0.64 (95% CI = 0.42-0.96). Super-Senior status also differed significantly by *APOE* haplotype using an overall model with a per allele OR of 0.76 (95% CI = 0.67-0.87, *p* = 0.00017). Compared to *APOEε3/3, APOEε2/2* was associated with increased odds for healthy aging, OR = 5.33 (95% CI = 1.55-18.34), and *APOEε3/4* and *APOEε2/4* did not reach significance against healthy aging with odds ratios of 0.71 (95% CI = 0.50-1.01) and 0.40 (95% CI = 0.16-1.00), respectively.

### Gene-gene interaction analysis

Among 7 previously reported gene-gene interactions, using an additive-additive model we did not observed any significant interactions (Table S2). The interaction term between rs6455128 in *KHDRBS2* (KH domain containing, RNA binding, signal transduction associated 2) and rs7989332 in *CRYL1* (crystallin lambda 1), however, was *p* = 0.077. Because the original rs6455128/rs7989332 interaction was found in a genome-wide association interaction analysis for AD, we adjusted for *APOEε4* carrier status and found that the p-value for the interaction decreased slightly (*p* = 0.061). Odds ratios for individual genotypes are shown in Table S3. Per genotype, it appears that there may be an interaction between *CRYL1* GG and *KHDRBS2* AC/AA.

Since most variants did not have a known interaction partner, we then tested for interactions between all combinations of the nine protective and eight deleterious variants (72 interaction tests) using an additive-additive model. Sex was included in all models. Three additional variant pairs showed evidence of interactions. *APOE* haplotype and rs9486902 in *FOXO3* (forkhead box O3) had a significant interaction (*p* = 0.035), as did rs10455872 in *LPA* (lipoprotein(a)) and rs7989332 CRYL1 (*p* = 0.041). *APOE* haplotype also showed evidence of an interaction with rs7989332 *CRYL1* (*p* = 0.049). No interactions withstood FDR correction. Odds ratios for individual genotypes in interacting pairs are shown in Tables S4-S6; due to low frequencies only *APOE4* carrier vs. non-carrier status, *CRYL1/LPA* and *CRYL1/KHDRBS2* dominant models are presented. There is some evidence that the APOE4 allele interacts with the rs9486902 *FOXO3* CC genotype, as well as the rs7989332 CRYL1 GG and GT genotypes.

**Table 4:** The top 20 functions and diseases represented in a candidate gene network in IPA®

Rank	Diseases and Functions
1	Disorder of lipid metabolism
2	Dyslipidemia
3	Concentration of sterol
4	Quantity of steroid
5	Concentration of triacylglycerol
6	Concentration of lipid
7	Atherosclerosis
8	Metabolism of triacylglycerol
9	Concentration of cholesterol
10	Hyperlipoproteinemia
11	Hyertriglyceridemia
12	Area of atherosclerotic lesion
13	Accumulation of lipid
14	Size of atherosclerotic lesion
15	Efflux of cholesterol
16	Homeostasis of lipid
17	Concentration of cholesterol ester
18	Hyperlipidemia
19	Dementia
20	Transport of lipid

There were also two pairs of SNPs with possible interactions. Rs1853021 in *LPA* and rs2802292 in *FOXO3* (*p* = 0.052) and rs1800562 in *HFE* (hemochromatosis) and rs56354395 in *ADIPOQ* (adiponectin, C1Q and collagen domain containing) (*p* = 0.059).

### Network analysis

**Figure 1:**
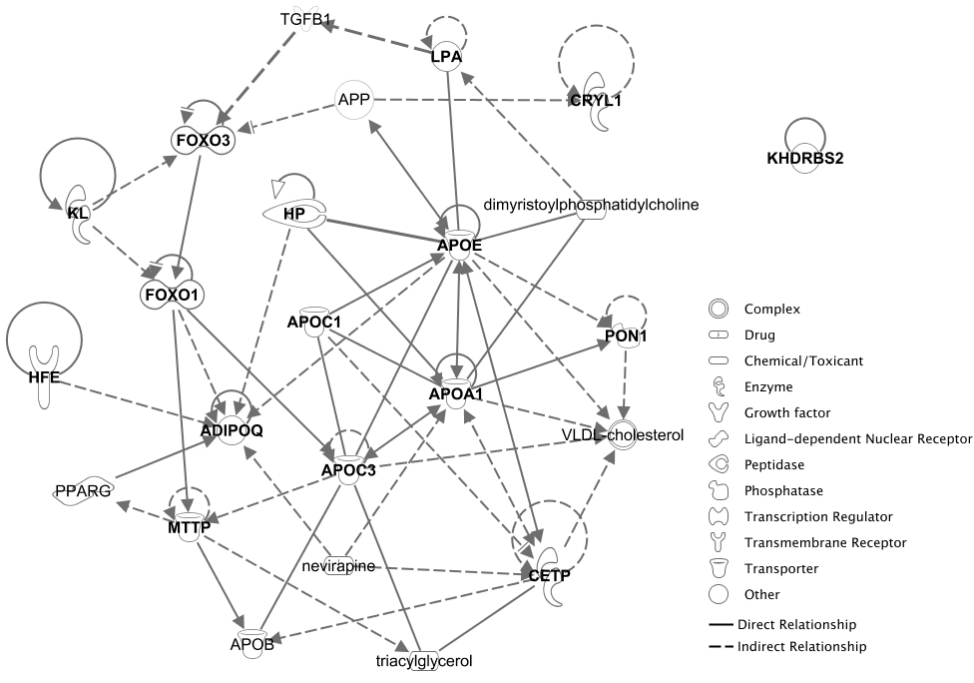
A network including 15 candidate epistatic longevity genes The diagram was created using QIAGEN's Ingenuity® Pathway Analysis software.

Network analysis was done using QIAGEN's Ingenuity^®^ Pathway Analysis (IPA^®^, QIAGEN Redwood City,www.qiagen.com/ingenuity) to characterize the types of genes identified during the literature search. Using the 15 genes from the literature search produced a network that connected 13 of the 15 query genes. The “grow” feature of IPA^®^ was used to expand the network to include additional molecules (Figure 1). When growing the network, priority is given to molecules that have the most overlap with the parts of the existing network that are the least connected. Of note, *KHDRBS2* and *CRYL1* did not have a known network connection with each other; however, *CRYL1* was connected to *APOE* by one node. *APOE* was also connected by one node to *FOXO3*, which was connected by one node to *LPA. HFE* and *ADIPOQ* were connected by a single edge.

The top functional and disease category represented in this network was metabolic disease, followed by hematological disease, lipid metabolism, and molecular transport. Multiple pathways are related to the genes in this network; the top 20 functions and diseases of those pathways are listed in Table 4; all of the top terms relate to lipids or cholesterol.

## DISCUSSION

*APOE* has three major alleles: *ε2* has been associated with decreased mortality [10, 11], *ε3* can be considered neutral, and *ε4* is associated with increased risk of AD and mortality [3]. Super-Seniors were less likely to carry an *APOEε4* allele, a finding that we previously published [12]. The *APOE ε2/2* diplotype was protective. A larger sample size would be needed to more confidently determine the effects of other diplotypes.

The *HP*2 allele contains a duplication of exons 3 and 4 of the haptoglobin gene [13], making the *HP1* and *HP2* alleles functionally different. Although the inverse association of *HP2* carrier status with healthy aging was not significant after FDR correction, it is consistent with the idea that the *HP*1/1 genotype is associated with longevity [14].

Several gene-gene interaction tests gave results that approached but did not achieve statistical significance. While we cannot reject the null hypothesis of no interaction in these cases, they represent candidate pairs with potentially intriguing biological roles that would be worth testing in other studies. One such pair is rs6455128 in *KHDRBS2* and rs7989332 in *CRYL1,* previously associated with AD [15]. Gusareva *et al*. hypothesized that the *CRYL1* encoded crystallin protein may act as a stress-protective heat-shock protein that could have a functional interaction with KHDRBS2, which also has a potential role in response to stress [15]. Gusareva *et al*. postulated that this interaction may occur within the TOR pathway [16], which influences β-amyloid plaques (Aβ) and AD-like deficits in a mouse model [17] and life span in model organisms [18, 19].

Some interactions between all combinations of variants were significant prior to multiple testing correction and may therefore be candidates for future replication analyses. *APOE* haplotype and rs9486902 in *FOXO3* showed an interaction effect. Per genotype, there may be an interaction between the *APOE4* allele and the *FOXO3 CC* genotype; *FOXO3 CC* could be a buffering genotype for the deleterious *APOE4*. Pathway analysis in IPA^®^ showed that one mechanism of interaction could be through amyloid beta precursor protein, APP. *FOXO3* is part of the insulin/insulin-like growth factor 1 signal pathway and has been associated with longevity [20], and FoxO proteins have been implicated in AD [21].

*APOE* haplotype and *LPA* rs10455872 had significant interaction effects with rs7989332 in *CRYL1*. The interaction between *APOE* and *CRYL1* may originate from an interaction between the *APOE4* allele and the *CRYL1* GG and GT genotypes. Interestingly, the *CRYL1* GG genotype also showed evidence of an interaction with the *KHDRBS2* AC/AA genotypes.

Another example is rs1853021 in *LPA*, which showed *p* = 0.052 for interaction with rs2802292 in *FOXO3*. Rs1853021 has been associated with elevated Lp(a) lipoprotein level, which is a risk factor for coronary disease, carotid atherosclerosis, and stroke [22]. Rs2802292 has been associated with longevity [20] and all-cause mortality [23].

Rs1800562 in *HFE* and rs56354395 in *ADIPOQ* (*p* = 0.059) were connected by a single edge in IPA^®^. The minor allele in *HFE* rs1800562 has been associated with risk of death, but has been seen to increase in frequency at older ages [24]. Increased serum adiponectin levels have been associated with longevity [25, 26]. Two variants in *ADIPOQ*, including rs56354395, have been associated with increased adiponectin levels and the del/del genotype had a higher prevalence in long-lived men [27].

When looking at the overall network, metabolic disease, hematological disease, lipid metabolism, and molecular transport were the most represented functional and disease categories. Despite the fact that many of the individual genes did not show significant differences in our population, it is interesting that lipid and cholesterol functions were significantly over-represented in the network. As well, a review of GWAS-identified risk genes for AD found that the associated genes clustered into three pathways: cholesterol and lipid metabolism, immune system and inflammatory response, and endosome vesicle cycling [28]. The idea that longevity is associated with a favourable lipid profile is not new. It has been found that individuals with exceptional longevity and their offspring have HDL and LDL particle sizes that are significantly larger than controls [29], that offspring of centenarians have favourable lipid profiles compared to their spouse controls [8], and that favourable HDL phenotypes and genotypes may contribute to a lower incidence of age-related diseases such as CVD and decreased mortality [30]. These results are all consistent with lipid and cholesterol maintenance being a key mediator in healthy aging and longevity.

Many of the candidate genes in our study were chosen in the literature reports by the original investigators due to their potential function in longevity. As a result, the selection of genes is biased; however, it is still valuable to examine themes, especially among the genes that were also significant in our study population, which represents long-term good health more than extreme longevity.

CVD and AD are age-related chronic diseases that decrease quality of life and increase risk of mortality. *APOEε4* confers a dose dependent increased risk for developing AD [3], and it was found in a meta-analysis that while the global frequency of the *ε4* allele is 13.7%, the allele frequency in AD patients is 36.7% [31]. *APOEε4* is also associated with hyperlipidemia and hypercholesterolemia, and causes neuroinflammation resulting in neurovascular dysfunction [32].

The two main neuropathological features seen in the brains of patients with AD are Aβ and neurofibrillary tangles [3]. ApoE is thought to help to remove Aβ from the brain by transporting it across the blood brain barrier; however, ApoE4 lipoproteins have a decreased binding affinity for Aβ compared to ApoE3 lipoproteins and may therefore be less efficient. ApoE also mediates delivery of cholesterol to neurons in the CNS, which is less efficient by ApoE4 than ApoE3 [32]. The CNS contains about 25% of total body cholesterol, which plays a key role in synaptic plasticity [33]. With age, there are system-wide changes in cholesterol metabolism, and this altered metabolism in the brain may relate to AD development [33]. There is also a decreased amount of cholesterol in the hippocampus and cortical areas in AD patients compared to age-matched controls [32].

Cardiovascular and neurovascular health share common risk factors including diabetes mellitus and hypertension [34]. Cognitively normal individuals with controlled hypertension have less Aβ accumulation than those with unmedicated hypertension. As well, the combination of carrying an *APOEε4* allele and having unmedicated hypertension increased the risk for Aβ accumulation [34].

Hp is an extracellular chaperone that acts as an antioxidant and anti-inflammatory by binding free hemoglobin, which it then transports to the liver [35]. Hp is produced in the brain in response to stress stimuli; it is increased in the cerebral spinal fluid of patients with AD and other neurodegenerative disorders [36]. Patients with AD consistently show signs of inflammation in their brains and oxidative stress is strongly implicated in AD etiology [35]. Hp has been found to be more oxidized in AD patients, and *in vitro*, oxidized Hp is less able to perform its chaperone function and inhibit Aβ aggregates [36, 37]. Aβ also competes with hemoglobin for binding to Hp, thus impairing its antioxidant function [36]. There is strong support that Aβ is central in AD pathogenesis and it is thought to trigger oxidative stress-mediated damage that leads to neuronal death [37].

*HP*1 and *HP*2 alleles form structurally different proteins that differ in hemoglobin binding and antioxidant capacity, and may be related to autoimmune and inflammatory disorders [38]. Despite the association of *HP*1/1 with longevity, there are conflicting results from studies looking at *HP* in relation to coronary heart disease (CHD) [39–41].

Our findings provide further evidence that *APOE* and genes in associated pathways are key players in healthy aging. This is consistent with a recent informed GWAS that utilized knowledge about age-related diseases to identify new extreme longevity loci that overlap with those associated with coronary artery disease and AD [42]. As well, in a whole genome sequencing study in a healthy aging cohort aged over 80 years, the Topol and Torkamani group found that healthy aging is associated with reduced genetic susceptibility to AD and coronary artery disease, but not cancer or diabetes [43]. In addition to *APOE* being the most replicable signal in GWAS of longevity, the search for more complex longevity haplotypes and interactions points towards mechanisms related to *APOE,* AD, and lipids.

Our results highlight pathways related to AD and reinforce the importance of lipids and cholesterol in healthy aging and longevity. Due to the exploratory nature of finding epistatic effects, it is unsurprising that the observed effects do not remain significant after multiple testing correction. However, these results are noteworthy as they represent additional candidates for buffering pairs that may be tested in other studies. The study of epistatic interactions, particularly buffering/ buffered pairs, is important as the identification of such pairs may help identify therapeutic drug targets for use in aiding individuals who do not carry health-protective longevity variants.

## MATERIALS AND METHODS

### Subjects

The current analysis included 466 Super-Seniors (female = 312, male = 154; mean = 88.6 years, SD = 3.0, range = 85-108 years), and 421 mid-life controls (female = 253, male = 168; mean = 46.8 years, SD = 3.3, range = 40-54 years) [9, 12]. The Super-Senior group included 140 subjects 90 years and older, 4 of whom were centenarians. Both groups of unrelated individuals were of European ancestry and lived in Metro Vancouver, British Columbia (BC), Canada. Controls were random and population-based, and recruited randomly from BC Medical Services Plan lists. Research ethics board approval was received from the joint Clinical Research Ethics Board of the BC Cancer Agency and the University of British Columbia and Simon Fraser University. All subjects gave written informed consent.

### Literature search

A literature search for protective buffering and deleterious buffered variants, as well as other epistatic effects associated with longevity was performed in PubMed. PubMed was chosen because of its biomedical and clinical focus. Search terms included combinations of: buffering, epistasis, aging, longevity, human, and genetics. Only variants found in human studies were considered. Variants located in the same gene were verified not to be in linkage disequilibrium at a threshold of r^2^ > 0.8.

### Genotyping

Sixteen SNPs were genotyped using Sequenom (San Diego, USA) iPLEX Gold technology at the McGill University and Genome Quebec Innovation Center. Two markers with a call rate below 95% were re-genotyped by the same method. 11 samples with a call rate < 90% across all markers were excluded. Three markers that could not be genotyped by the Sequenom method were either replaced by another marker in linkage disequilibrium (rs2542052 in *LPA*) or genotyped by TaqMan^®^ (rs56354395 in *ADIPOQ*) or PCR (rs72294371 in *HP*). Custom TaqMan^®^ probes were designed using the Thermo Fisher Scientific (Waltham, USA) online tool (www.thermofisher.com).

A 1724 bp insertion in *HP* was genotyped by PCR using a two primer design as described by Koch *et al*. [13]; products were sized on an agarose gel. The first primer set: 5′-AGCCCACCCCTCCACCTATGTGCC-3′ and 5′-GCTTAAGATCCCAGTCGCATACC-3′ [44], yielded a 3221 or 4945bp product, corresponding to the *HP1* allele and *HP2* allele, respectively. Because the larger *HP2* product was not always clearly visible when the gel was imaged, a second set of primers was used to detect this allele. The second primer set: 5′-CCCAGCCTCTTCTGCTCTTA-3′ and 5′-TGCACATCAATCTCCTTCCA-3′ yielded a 248bp product only when the *HP*2 allele was present.

### Association tests of individual variants

Analyses were performed in R 3.2.2. Individual variants were tested using logistic regression to estimate odds ratios and 95% confidence intervals for associations between Super-Senior status and variants. Super-Seniors and controls were coded as 1 and 0. Models were adjusted for sex. Dominant and additive models were tested. In the dominant model, the major allele homozygote was coded as 0, and the heterozygote and minor allele homozygote were both coded as 1. Exceptions to this were *APOE* and *HP,* which were coded for the presence of carrying the risk-associated *APOEε4* allele and *HP*2 allele, respectively. In the additive model, genotypes were coded as 0, 1, 2. All *p* values were determined using the likelihood ratio test. The false discovery rate (FRD threshold = 0.05) was used to adjust for multiple comparisons.

### Gene-gene interaction analysis

Gene-gene interactions were tested using an additive-additive model. Logistic regression analysis was conducted as follows: *y ∼ variant1 + variant2 + variant1 x variant2 + sex* (function = glm, family = binomial, link = logit). Super-Senior/control status was the outcome variable.

First, the 7 epistatic pairs from the literature were independently tested to see if they were observed in our population. Then all combinations of putative protective and deleterious variants were compared. FDR was used to adjust for multiple comparisons.

### Network analysis

Pathway analysis was conducted using QIAGEN Ingenuity^®^ Pathway Analysis (IPA^®^, QIAGEN Redwood City,www.qiagen.com/ingenuity) to characterize the types of genes identified during the literature search. IPA^®^ uses the curated Ingenuity^®^ Knowledge Base constructed from peer-reviewed journals and biomedical databases to construct networks of connections between genes and molecules. The 15 genes from the literature search were entered into IPA^®^ to produce a network that was then “grown” to include additional related molecules. IPA^®^ was also used to identify functions and diseases that were most significantly represented in the network.

## SUPPLEMENTARY MATERIALS FIGURES


