# Supplementary material for: Lipid and Alzheimer’s disease genes associated with healthy aging and longevity in healthy oldest-old
Source: Oncotarget. 2017 Feb 11;8(13):20612–21. doi: 10.18632/oncotarget.15296 (PMC5400530; doi:10.18632/oncotarget.15296)
Supplement: Supplementary file 1 [file oncotarget-08-20612-s001.pdf]

Lipid and Alzheimer's disease genes associated with healthy aging and longevity in healthy oldest-old

Supplementary Material

Supplementary Table 1: *p*-values for main effects examined

Gene	ID	Dominant model *p* value (df)	Additive model *p* value (df)
ADIPOQ	rs56354395	0.50 (1)	0.24 (1)
APOA1	rs670	0.85 (1)	0.88 (1)
APOC3	rs595049	0.61 (1)	0.88
APOE	*APOE* haplotype	0.0010 (1)*	0.00017 (5)*
CETP	rs5882	0.54 (1)	0.36 (1)
CRYL1	rs7989332	0.88 (1)	0.96 (1)
FOXO1	rs2701858	0.089 (1)	0.11 (1)
FOXO3	rs9486902	0.57 (1)	0.60 (1)
FOXO3	rs2802292	0.16 (1)	0.24 (1)
HFE	rs1800562	0.32 (1)	0.34 (1)
HP	rs72294371	0.010 (1)*	0.056 (1)
KHDRBS2	rs6455128	0.29 (1)	0.48 (1)
KL	rs9536314	0.12 (1)	0.070 (1)
LPA	rs1853021	0.58 (1)	0.99 (1)
LPA	rs10455872	0.31 (1)	0.25 (1)
MTTP	rs2866164	0.34 (1)	0.53 (1)
PON1	rs662	0.72 (1)	0.93 (1)

Supplementary Table 2. *p*-values for interaction tests identified a priori

Variant 1	Variant 2	*p* value (df)
CRYL1 rs798933	*KHDRBS2* rs6455128	0.077 (1)
FOXO3 rs9486902	*FOXO1* rs2701858	0.38 (1)
LPA rs1853021	*CETP* rs5882	0.63 (1)
MTTP rs2866164	*CETP* rs5882	0.57 (1)
MTTP rs2866164	*APOC3* rs595049	0.42 (1)
MTTP rs2866164	*ADIPOQ* rs56354395	0.10 (1)
APOE haplotype	*HP* rs72294371	0.37 (1)

Supplementary Table3. Interaction odds ratios and 95% confidence intervals between *KHDRBS2* rs6455128 and *CRYL1* rs7989332

<table>
<tr><td></td><td></td><td colspan="2">CRYL1 rs7989332</td></tr>
<tr><td rowspan="3">KHDRBS2 rs6455128</td><td></td><td>GG</td><td>GT or TT</td></tr>
<tr><td>CC</td><td>1.0</td><td>0.80 (0.58-1.11)</td></tr>
<tr><td>AC or AA</td><td>0.64 (0.43-0.94)*</td><td>0.96 (0.64-1.46)</td></tr>
</table>

Supplementary Table 4. Interaction odds ratios and 95% confidence intervals between *APOE4* carrier status and *FOXO3* rs948602

		APOE4 carrier	
FOXO3 rs948602		Non-carrier	Carrier
	CC	1.0	0.51 (0.35-0.73)*
	CT	0.83 (0.58-1.20)	0.70 (0.36-1.35)
	TT	0.61 (0.25-1.5)	2.71 (0.3-24.5)

Supplementary Table 5. Interaction odds ratios and 95% confidence intervals between *CRYL1* rs7989332 and *LPA* rs10455872

		CRYL1 rs7989332	
LPA rs10455872		GG	GT or TT
	TT	1.0	1.07 (0.80-1.42)
	CT or CC	1.09 (0.66-1.81)	0.54 (0.28-1.03)

Supplementary Table 6. Interaction odds ratios and 95% confidence intervals between *APOE4* carrier status and *CRYL1* rs7989332

		APOE4 carrier	
CRYL1 rs7989332		Non-carrier	Carrier
	GG	1.0	0.50 (0.32-0.76)*
	GT	0.89 (0.64-1.22)	0.53 (0.32-0.90)*
	TT	0.76 (0.39-1.48)	1.53 (0.51-4.59)
